# The Impact of Surveillance Imaging Frequency on the Detection of Distant Disease for Patients with Resected Stage III Melanoma

**DOI:** 10.1245/s10434-021-11231-3

**Published:** 2022-02-10

**Authors:** Mbathio Dieng, Sarah J. Lord, Robin M. Turner, Omgo E. Nieweg, Alexander M. Menzies, Robyn P. M. Saw, Andrew J. Einstein, Louise Emmett, John F. Thompson, Serigne N. Lo, Rachael L. Morton

**Affiliations:** 1 NHMRC Clinical Trials Centre, Faculty of Medicine and Health, The University of Sydney, Camperdown, NSW Australia; 2 Centre for Biostatistics, University of Otago, Dunedin, New Zealand; 3 Melanoma Institute Australia, The University of Sydney, Sydney, NSW Australia; 4 Faculty of Medicine and Health, Sydney Medical School, The University of Sydney, Sydney, NSW Australia; 5 Department of Melanoma and Surgical Oncology, Royal Prince Alfred Hospital and Mater Hospitals, Sydney, NSW Australia; 6 Seymour, Paul, and Gloria Milstein Division of Cardiology, Department of Medicine, and Department of Radiology, Columbia University Irving Medical Center and New York-Presbyterian Hospital, New York, NY USA; 7 Department of Theranostics and Nuclear Medicine, St Vincent’s Hospital Sydney, Sydney, NSW Australia; 8 Department of Medical Oncology, Royal North Shore and Mater Hospitals, Sydney, NSW Australia

## Abstract

**Background:**

It is not known whether there is a survival benefit associated with more frequent surveillance imaging in patients with resected American Joint Committee on Cancer stage III melanoma.

**Objective:**

The aim of this study was to investigate distant disease-free survival (DDFS), melanoma-specific survival (MSS), post distant recurrence MSS (dMSS), and overall survival for patients with resected stage III melanoma undergoing regular computed tomography (CT) or positron emission tomography (PET)/CT surveillance imaging at different intervals.

**Patients and Methods:**

A closely followed longitudinal cohort of patients with resected stage IIIA–D disease treated at a tertiary referral center underwent 3- to 4-monthly, 6-monthly, or 12-monthly surveillance imaging between 2000 and 2017. Survival outcomes were estimated using the Kaplan–Meier method, and log-rank tests assessed the significance of survival differences between imaging frequency groups.

**Results:**

Of 473 patients (IIIA, 19%; IIIB, 31%; IIIC, 49%; IIID, 1%) 30% underwent 3- to 4-monthly imaging, 10% underwent 6-monthly imaging, and 60% underwent 12-monthly imaging. After a median follow-up of 6.2 years, distant recurrence was recorded in 252 patients (53%), with 40% detected by surveillance CT or PET/CT, 43% detected clinically, and 17% with another imaging modality. Median DDFS was 5.1 years (95% confidence interval 3.9–6.6). Among 139 IIIC patients who developed distant disease, the median dMSS was 4.4 months shorter in those who underwent 3- to 4-monthly imaging than those who underwent 12-monthly imaging.

**Conclusion:**

Selecting patients at higher risk of distant recurrence for more frequent surveillance imaging yields a higher proportion of imaging-detected distant recurrences but is not associated with improved survival. A randomized comparison of low versus high frequency imaging is needed.

**Supplementary Information:**

The online version contains supplementary material available at 10.1245/s10434-021-11231-3.

Around half of patients with resected stage III melanoma will develop a recurrence, with the risk dependent on substage.^1,2^ Approximately 50% of recurrences will be in regional lymph nodes and 30% will be at distant sites.^3^ Although various imaging modalities are widely used with the objective of detecting recurrences at an early stage, there is considerable variability in the frequency of surveillance imaging, as it is not clear whether earlier detection of metastatic disease, facilitating earlier treatment, translates into survival benefits. In the absence of randomized trials, current clinical practice guidelines provide conflicting recommendations for the frequency and duration of surveillance imaging.^4–7^

Since 2011, potentially effective targeted drug therapies and immunotherapies have improved the survival of patients with stage IV (distant) melanoma. This has generated impetus to identify and treat metastatic spread as early as possible.^8–10^ In addition, adjuvant systemic therapy is administered to patients with resected high-risk stage III disease to prevent recurrence. Thus, the role of imaging is increasingly relevant and several randomized trial protocols of adjuvant treatment for patients with resected stage III melanoma have required imaging every 12 weeks.^11,12^

Without randomized trials to estimate any survival benefit of earlier versus later detection and treatment of distant recurrence, clinical guideline recommendations for imaging surveillance frequency must rely on observational studies. Critical questions that may be addressed by observational studies include the incidence of distant disease, methods of detection of that disease, time to distant disease, and disease-specific and overall survival (OS). This evidence will also be valuable to inform the development of decision models to estimate the efficacy and cost effectiveness of surveillance imaging and the design of definitive clinical trials.^13^

The aims of this study were to determine the proportion of patients with resected stage III melanoma who developed distant recurrence identified with computed tomography (CT) or positron emission tomography (PET)/CT surveillance imaging over a period of at least 5 years, by tumor substage and screening interval. Secondary objectives were to describe distant disease-free survival (DDFS), melanoma-specific survival (MSS), post distant recurrence MSS (dMSS), and OS by surveillance interval for each group.

## Methods

### Study Design and Data Sources

This retrospective cohort study was conducted using the Melanoma Institute Australia (MIA) database and clinical trial files. The MIA database is one of the world’s largest prospective clinical research databases of melanoma patients (*n* = 60,000), with carefully documented cases containing a complete medical history, all investigations, treatments, and survival status. Characteristics of the primary melanoma, substage at the time of diagnosis of stage III disease (IIIA–D), the number and type of scans performed until distant disease recurrence, time to recurrence, and disease management for recurrence were extracted from patients’ medical records. The frequency of the imaging schedule was determined by clinician preference and was grouped as 3- to 4-monthly, 6-monthly, or 12-monthly. Survival status and deaths due to melanoma were confirmed using linkage to the National Death Index.^14^

### Patient Cohort

At MIA, 473 consecutive melanoma patients with resected American Joint Committee on Cancer (AJCC) 8th edition^15^ stage IIIA–D disease were identified in an imaging program, between 2000 and 2017. These patients underwent repeated surveillance CT or PET/CT imaging as part of their scheduled long-term follow-up.

### Inclusion Criteria

We included asymptomatic stage IIIA–D melanoma patients who completed definitive surgical treatment and underwent a baseline CT or PET/CT plus at least one follow-up scan (3–12 months post-surgery) that showed no evidence of disease. Patients participating in a clinical trial who had surveillance imaging according to their trial protocol were also included.

### Exclusion Criteria

Patients were excluded from the cohort if they had no baseline imaging test or were diagnosed with distant disease prior to their first scheduled surveillance imaging test (i.e. within 6–12 months of surgery).

All patients were followed until a confirmed diagnosis of distant metastatic disease, death, end of surveillance schedule, or loss to follow-up.

### Outcomes

The key outcomes were the proportion of patients diagnosed with distant recurrence as a result of surveillance imaging, DDFS, MSS, post dMSS, and OS (electronic supplementary Fig. 1). For this study, only results from the routinely scheduled surveillance CT or PET/CT imaging were included. Patients were grouped by the interval selected at the beginning of the surveillance period. Clinicians may have ordered further imaging on the basis of patients’ symptoms or findings of the routine tests; these were considered as ‘extra investigations’ and not part of the routine schedule.

DDFS was defined as the time from diagnosis of stage III melanoma to first reported distant recurrence, or melanoma-related death; MSS was defined as the time from stage III diagnosis to death from melanoma; dMSS was defined as the time from stage IV diagnosis to melanoma-related death; and OS was defined as the time from stage III diagnosis to death from any cause.

### Statistical Analysis

The clinical and histopathological characteristics, as well as the proportion of recurrences detected by imaging, were summarized using descriptive statistics. All survival outcomes were described using the Kaplan–Meier method, along with the estimated 2- and 5-year survival rates, median survival, and 95% confidence intervals (CIs). The log-rank test was used to assess survival differences between imaging frequency groups. Subgroup analyses were run to assess differences in survival outcomes (OS, MSS, and DDFS) by imaging frequency within each AJCC 8th edition substage. Univariate Cox proportional hazards regression models determined the association of clinical characteristics with MSS and OS. A multivariable Cox proportional hazards model stratified by substage for MSS and OS was obtained by first including all covariates with a *p*-value < 0.25 from univariate regression and then performing a stepwise backward selection using a threshold of 0.05 for the significance level. As supplementary analysis, we further investigated if patients diagnosed before and after 2010 had different clinicopathological features and then compared the survival for those two diagnosis periods. All analyses were performed using R version 3.6.1 (The R Foundation for Statistical Computing, Vienna, Austria) and SAS version 9.4 (SAS Institute, Cary, NC, USA).

The study was reviewed and approved by the MIA research committee (#MIA2016/182). All participants had given consent for their clinical and histopathological data to be collected in the MIA database and used for research. Ethical approval was granted to MIA by the Royal Prince Alfred Hospital Health Research Ethics Committee (# X15-0311 [previously X10-0300] and HREC/10/RPAH/530) for the use of these de-identified data in retrospective research studies.

## Results

### Demographic Characteristics

A total of 1394 patients with stage III disease treated and followed-up between 2000 and 2017 were identified. Of these, 540 did not have any imaging recorded in the MIA database and were therefore excluded. An additional 381 patients underwent intermittent or ad hoc imaging that could not be categorized into one of the three schedules and these patients were also excluded. Of 473 patients meeting all eligibility criteria and included in the study cohort, the mean age was 54 years, 64% were males, and 50% were AJCC 8th edition stage IIIC or higher (Table 1). The median duration of follow up was 6.2 years (95% CI 6.0–6.4 years). Imaging was performed 3- to 4-monthly in 30% of the patients, 6-monthly in 10% of patients, and 12-monthly in 60% of patients. CT was the most frequently used imaging modality (for 85% of patients) versus PET/CT (15%). For participants undergoing 3- to 4-monthly imaging, 66% were stage IIIC or IIID; for those undergoing 6-monthly imaging, 62% were stage IIIC or IIID; and 41% of those who underwent 12-monthly imaging were stage IIIC or IIID.

**Table 1:** Patient characteristics

Characteristics	All patients [*N* = 473]	12-monthly [*n* = 285]	6-monthly [*n* = 47]	3- to 4-monthly [*n* = 141]	*p*-Value
*Age at stage III diagnosis, years*					
Mean (SD)	54.4 (14.8)	52.0 (14.7)	53.7 (13.7)	59.7 (14.2)	< 0.001
Median (range)	56 (19–89)	53 (19–89)	55 (21–76)	60 (26–89)	
*Sex*					
Male	303 (64.1)	182 (63.9)	33 (70.2)	88 (62.4)	0.624
Female	170 (35.9)	103 (36.1)	14 (29.8)	53 (37.6)	
*Primary site*					
Head and neck	74 (15.6)	43 (15.1)	9 (19.1)	22 (15.6)	0.003
Trunk	150 (31.7)	102 (35.8)	12 (25.5)	36 (25.5)	
Upper limb	50 (10.6)	33 (11.6)	4 (8.5)	13 (9.2)	
Lower limb	113 (23.9)	73 (25.6)	12 (25.5)	28 (19.9)	
Occult	86 (18.2)	34 (11.9)	10 (21.3)	42 (29.8)	
*AJCC 8th edition stage*					
IIIa	89 (18.8)	78 (27.4) [88%]	6 (12.8) [7%]	5 (3.5) [5%]	< 0.001
IIIb	146 (30.9)	91 (31.9) [62%]	12 (25.5) [8%]	43 (30.5) [29%]	
IIIc	231 (48.8)	113(39.6) [49%]	28 (59.6) [12%]	90 (63.8) [39%]	
IIId	7 (1.5)	3 (1.1) [43%]	1 (2.1) [14%]	3 (2.1) [43%]	
*Breslow thickness* (mm)					
Mean (SD)	3.9 (4.0)	3.3 (3.6)	5.4 (6.1)	4.8 (3.8)	< 0.001
Median (range)	2.7 (0.0–40.0)	2.3 (0.5–40.0)	3.5 (1.0–27.5)	3.7 (0.0–21.0)	
*Mitotic rate*					
Mean (SD)	7.3 (7.3)	6.8 (6.2)	7.8 (10.8)	8.3 (8.2)	0.196
Median (range)	5.0 (0.0–57.0)	5.0 (0.0–45.0)	4.0 (0.0–49.0)	6.0 (0.0–57.0)	
*Ulceration*					
No	221 (58.8)	159 (64.4)	18 (51.4)	44 (46.8)	0.009
Yes	155 (41.2)	88 (35.6)	17 (48.6)	50 (53.2)	
*Regression*					
Absent	134 (34.6)	80 (31.9)	14 (37.8)	40 (40.4)	0.305
Early	177 (45.7)	124 (49.4)	14 (37.8)	39 (39.4)	
Intermediate	20 (5.2)	14 (5.6)	0 (0.0)	6 (6.1)	
Late	33 (8.5)	21 (8.4)	5 (13.5)	7 (7.1)	
Not reported	23 (5.9)	12 (4.8)	4 (10.8)	7 (7.1)	

The values in brackets represent percentage

Data are expressed as *n* (%) unless otherwise specified

*SD* standard deviation, *AJCC* American Joint Committee on Cancer

### Distant Recurrence

**Fig. 1:**
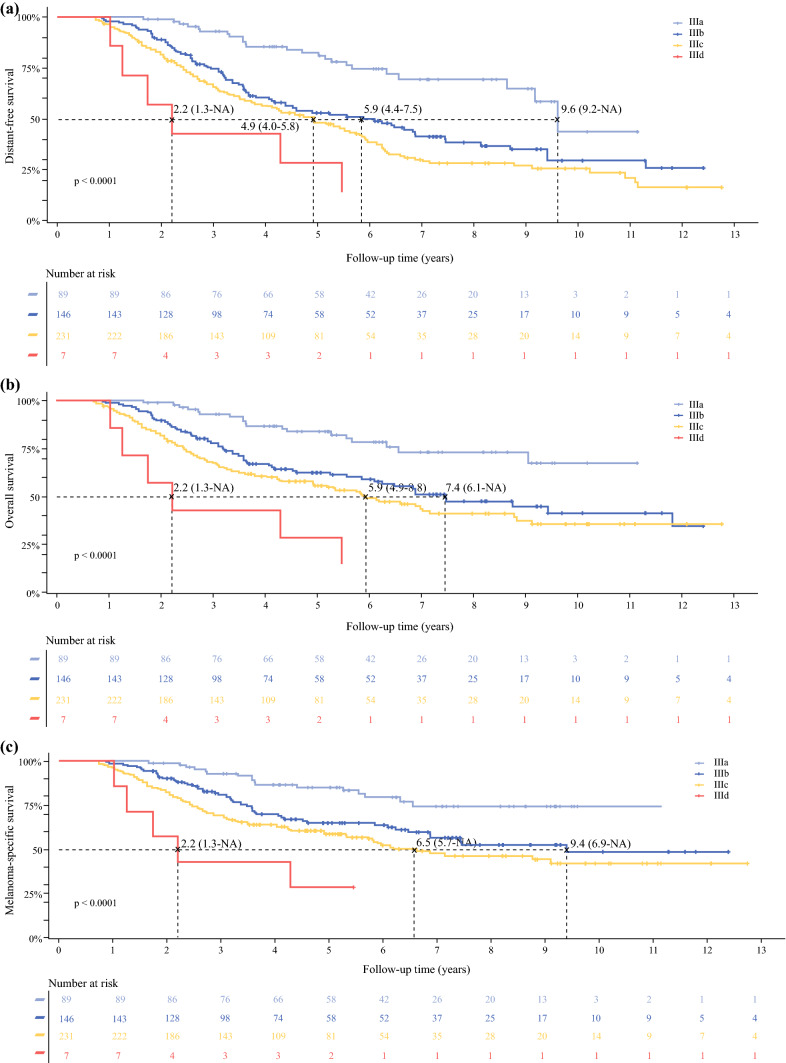
**a** Distant disease-free survival, **b** overall survival, and **c** melanoma-specific survival by melanoma substage. *NA* not available

Distant recurrence was detected in 252 patients (53%) (Table 2). The median DDFS was 5.1 years (95% CI 3.9–6.6) and varied by substage (Fig. 1). The cumulative incidence of distant recurrence at 2 and 5 years ranged from 10 to 52% for stage IIIA–B, to 42% and 87% for Stage IIIC–D, respectively (Fig. 1).

**Table 2:** Mode of detection of distant recurrence

	Stage								
	IIIa (*n* = 24)			IIIb (*n* = 83)			IIIc (*n* = 139)		
	3-monthly imaging (%)	6-monthly imaging (%)	12-monthly imaging (%)	3-monthly imaging (%)	6-monthly imaging (%)	12-monthly imaging (%)	3-monthly imaging (%)	6-monthly imaging (%)	12-monthly imaging (%)
Routine imaging (CT, PET/CT)	20	100	41	43	67	37	46	29	32
Clinical (reactive) [then confirmed by FNAB, cytology, histopathology or hematology]	80	–	59	57	33	63	54	71	68

*CT* computed tomography, *PET* positron emission tomography, *FNAB* fine-needle aspiration biopsy

**Fig. 2:**
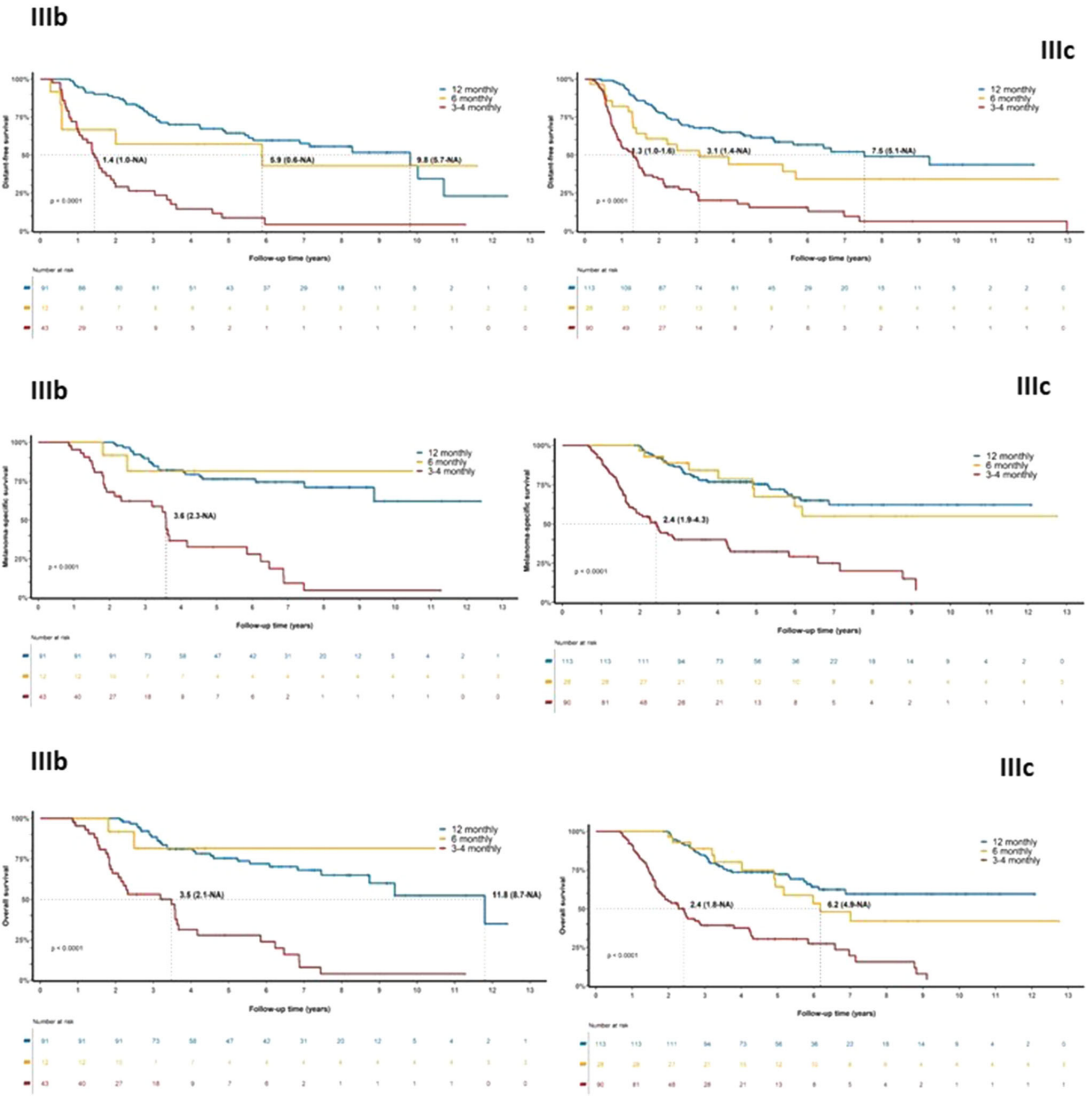
**a** Distant disease-free survival, **b** melanoma-specific survival, and **c** overall survival by imaging frequency for American Joint Committee on Cancer stages IIIB and IIIC, from the time of stage III diagnosis. *NA* not available

Forty percent of the distant recurrences were first detected by CT or PET/CT; 43% were detected by clinical examination (then confirmed with fine-needle aspiration biopsy, cytology or histopathology), and 17% were detected by another imaging modality such as magnetic resonance imaging, plain x-ray, or ultrasound. For patients with stage IIIC disease, 46% of distant recurrences were detected by CT or PET/CT in the 3- to 4-monthly imaging group compared with 32% in the 12-monthly imaging group (Table 2). The cumulative incidence of distant recurrences was 33% at 2 years and 50% at 5 years. Within substages IIIB and IIIC, median DDFS was shorter in those receiving 3- to 4-monthly imaging than those receiving 6-monthly or 12-monthly imaging (*p* < 0.0001) (Fig. 2).

### Survival

**Fig. 3:**
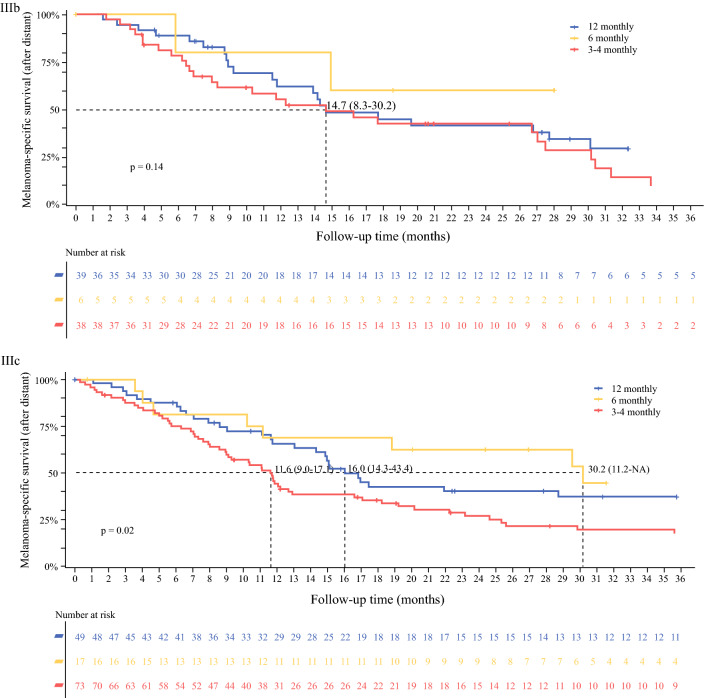
Post distant melanoma-specific survival by imaging frequency for American Joint Committee on Cancer stages IIIB and IIIC, from the time of distant melanoma diagnosis

The median MSS time for the whole cohort was 14 years (95% CI 7.2 to not reached). Within substages IIIB and IIIC, the median MSS was shorter in those receiving 3- to 4-monthly imaging than in those receiving 6-monthly or 12-monthly imaging (*p* < 0.0001) (Fig. 2). Among the 252 patients who developed distant recurrence, median post dMSS was 3.6 years (95% CI 3.1–4.1 years). Of these patients, 55% (*n* = 139) were stage IIIC and 33% were stage IIIB (Fig. 3). For the 139 patients with stage IIIC disease who developed distant recurrence, the median post dMSS was 11.6 months (95% CI 9.0–17.1 months) in the 3- to 4-monthly group and 16 months (95% CI 14.3–43.4 months) in the 12-monthly imaging group. For patients with stage IIIB disease who developed distant recurrence, the median post dMSS in the 3- to 4-monthly and 12-monthly imaging groups was the same, i.e. 14.7 months (95% CI 8.3–30.2 months).

The median OS time for the whole cohort was 7.4 years (95% CI 6.5–11.8). Within substages IIIB and IIIC, the median OS was shorter in those receiving 3- to 4-monthly imaging than in those receiving 6-monthly or 12-monthly imaging (*p* < 0.0001) (Fig. 2).

### Associations Between Clinical Parameters and Survival

Associations between clinical parameters and survival outcomes are summarized in Table 3. In multivariable analysis, for MSS, after stratifying by substage, higher Breslow thickness (hazard ratio [HR] 1.06, 95% CI 1.01–1.09, *p* = 0.002) and the 3- to 4-monthly imaging schedule (HR 5.16, 95% CI 3.54–7.52, *p* < 0.001) were associated with lower survival (electronic supplementary Table 1). Similarly, for OS, higher Breslow thickness (HR 1.05, 95% CI 1.02–1.09, *p* = 0.003) and a 3- to 4-monthly imaging schedule (HR 5.08, 95% CI 3.53–7.32, *p* < 0.001) were associated with lower OS (electronic supplementary Table 2).

**Table 3:** Pooled baseline characteristics stratified by whether or not patients had a distant recurrence during the follow-up period

Characteristics	All patients [*N* = 473]	No-distant [*n* = 221]	Distant [*n* = 252]	*p*-Value
*Type of FU schedule*				
12-monthly	285 (60.3)	176 (79.6)	109 (43.3)	< 0.0001
6-monthly	47 ( 9.9)	23 (10.4)	24 ( 9.5)	
3- to 4-monthly	141 (29.8)	22 (10.0)	119 (47.2)	
*AJCC 8th Edition stage*				
IIIa	89 (18.8)	65 (29.4)	24 (9.5)	< 0.0001
IIIb	146 (30.9)	63 (28.5)	83 (32.9)	
IIIc	231 (48.8)	92 (41.6)	139 (55.2)	
IIId	7 ( 1.5)	1 ( 0.5)	6 (2.4)	
*Age at stage III diagnosis, years*				
Mean (SD)	54.4 (14.8)	52.0 (15.4)	56.6 (13.9)	0.0008
Median (range)	56.0 (19.0–89.0)	53.0 (19.0–89.0)	57.0 (20.0–89.0)	
*Sex*				
Male	303 (64.1)	142 (64.3)	161 (63.9)	0.9343
Female	170 (35.9)	79 (35.7)	91 (36.1)	
*Primary site*				
Head and neck	74 (15.6)	30 (13.6)	44 (17.5)	0.1330
Trunk	150 (31.7)	76 (34.4)	74 (29.4)	
Upper limbs	50 (10.6)	23 (10.4)	27 (10.7)	
Lower limbs	113 (23.9)	60 (27.1)	53 (21.0)	
Occult	86 (18.2)	32 (14.5)	54 (21.4)	
*Breslow thickness* (mm)				
Mean (SD)	3.9 (4.0)	3.1 (3.4)	4.7 (4.4)	< 0.0001
Median (range)	2.7 (0.0–40.0)	2.2 (0.5–40.0)	3.5 (0.0–28.0)	
*Mitotic rate*				
Mean (SD)	7.3 (7.3)	6.0 (6.2)	8.5 (8.0)	0.0011
Median (range)	5.0 (0.0–57.0)	4.0 (0.0–45.0)	6.0 (0.0–57.0)	
*Ulceration*				
No	221 (58.8)	123 (66.8)	98 (51.0)	0.0019
Yes	155 (41.2)	61 (33.2)	94 (49.0)	
*Regression*				
Absent	134 (34.6)	58 (30.7)	76 (38.4)	0.2961
Early	177 (45.7)	88 (46.6)	89 (44.9)	
Intermediate	20 (5.2)	11 (5.8)	9 ( 4.5)	
Late	33 (8.5)	21 (11.1)	12 (6.1)	
Not reported	23 (5.9)	11 (5.8)	12 (6.1)	

Data are expressed as *n *(%) unless otherwise specified

*FU* follow-up, *AJCC* American Joint Committee on Cancer, *SD* standard deviation

The results of the analysis of survival by time of diagnosis are provided in electronic supplementary Table 4 and electronic supplementary Fig. 2. Patients diagnosed after 2010 were more likely to undergo a more intensive follow-up schedule. In addition, DDFS was significantly different for the two time periods, but no significant difference was observed for OS and MSS.

## Discussion

Our findings from a closely followed cohort of 473 patients with resected stage III melanoma provide valuable evidence about the clinical outcomes of patients undergoing routine CT or PET/CT at 3- to 4-monthly, 6-monthly, and 12-monthly intervals. In this patient group with a high rate of distant recurrence, surveillance CT and PET/CT imaging detected a high proportion of unsuspected distant recurrences. For stage IIIC, the proportion of patients whose distant recurrence was detected by surveillance imaging was 14% higher in the 3- to 4-monthly group than in the 12-monthly imaging group; however, we did not find evidence of improved survival. Patients who underwent 3- to 4-monthly imaging had poorer prognostic characteristics than those who underwent 12-monthly imaging, with the impact of this evidenced in their median MSS and OS.

The proportion of patients with distant disease detected by CT or PET/CT imaging in our follow-up cohort was higher than reported in some previous studies. One study, in which patients with stage III melanoma were examined every 3 months for 5 years after surgery, and in which imaging was used to investigate findings suspicious for recurrence, reported 28% of distant metastases were detected by CT imaging.^16^ Another study, with 3-monthly imaging for the first 2 years then 6-monthly imaging, reported 32% of recurrences were detected by radiography, largely CT scans (72%).^1^ However our recurrence detection rates were lower than those reported in other studies. For example, one study reported 68% of recurrences were detected through surveillance imaging (6-monthly intervals),^17^ while another study undertaken in asymptomatic patients reported 75% of systemic recurrences were detected by CT imaging.^18^ It is possible that imaging-detected metastases were also visible to the naked eye or clinically palpable and would have been identified by physical examination. In our study, these were classified as having been detected by imaging, but may have been classified as detected by physical examination if the clinical consultation occurred before the imaging.

Despite a greater proportion of substage IIIB and IIIC patients in our cohort, the DDFS of our cohort of 61.2 months was substantially longer than the DDFS in a cohort of 870 stage III patients undergoing 6-monthly imaging (21.2 months), and longer than the DDFS in the placebo arm of an adjuvant dabrafenib and trametinib trial (16.6 months) containing 870 stage III patients, and the DDFS reported in the adjuvant ipilimumab trial (17.1 months) containing 951 stage III patients.^11,19,20^

In our study, MSS, identification of post dMSS and OS were shorter for the more frequent imaging group; however, this is likely to reflect the characteristics of patients who were followed-up more frequently. The majority of patients in the more frequent follow-up group were stage IIIC (64%), compared with 40% in the 12-monthly imaging group. If the main proposed benefit of more frequent surveillance is to improve dMSS, then one would expect to see a longer dMSS in the more frequently imaged group than the less frequently imaged group within a given substage. Our data for stage IIIB and IIIC patients showed no improvement, which highlights the equipoise in these surveillance regimens and strongly supports the need for a prospective randomized trial.

In addition, the majority of our cohort was treated before the era of adjuvant therapies, therefore our results may not be directly applicable to the current situation, where such therapies are used with increasing frequency. Adjuvant therapy for melanoma in stage III patients has shown efficacy in terms of both DDFS and OS.^19,21,22^ In our cohort of stage III patients, a total of 26 (5%) received adjuvant therapy. Of these, 13 patients were receiving adjuvant interferon, 2 patients were receiving neoadjuvant therapy, 9 patients were receiving targeted therapy, and 2 patients were receiving immune therapy. The low rate of treatment with adjuvant immunotherapy in this cohort reflects the time period of the study cohort (2000–2017). Adjuvant immunotherapy was only available outside of clinical trials (Therapeutic Goods Administration [TGA]-approved) in March 2018, and was not affordable for many patients until it was subsidized by the Pharmaceutical Benefits Scheme (PBS) in November 2019. Furthermore, adjuvant immunotherapy was most commonly prescribed for stage III patients who had recently completed surgical treatment, whereas many of our cohort had been in post-surgical follow-up for many years by the time the drugs were publicly available. With adjuvant therapy, regular imaging may be required to assess the efficacy of the drug regimen and to identify or confirm toxicities such as pneumonitis. However, limited data are available regarding the optimal surveillance imaging strategy for asymptomatic stage III melanoma patients during and after a course of postoperative adjuvant therapy.

Whether more frequent surveillance imaging improves survival through earlier detection remains to be clarified. Therefore, in the absence of randomized trials, different imaging frequencies with their associated additional radiation exposure, increased financial costs and ‘scanxiety’ need to be carefully considered. Findings from this study will be used to populate a decision model assessing the cost effectiveness of the three imaging frequencies compared with no routine imaging. The proportion of distant recurrences detected by each imaging strategy, the survival after a distant recurrence, and distant recurrence detection rates are valuable inputs for decision models. This study provides estimates of eligible patient numbers, the potential health outcomes such as survival after a distant recurrence, the minimal clinically important differences, and actual clinician practice patterns. This information provides necessary insights into trial feasibility and helps determine the sample size and the target population, and establishes the feasibility of the study protocol. Furthermore, these survival data and their precision estimates can be used to populate cost-effectiveness models to estimate the costs and benefits of different surveillance imaging strategies for stage III melanoma patients.

There were some limitations to our study. First, the comparison of the three imaging frequency schedules was not randomized, introducing some selection bias with more frequent imaging schedules recommended for patients at higher risk of recurrence. Second, the analysis was conducted retrospectively. Although the MIA database is the world’s largest prospective clinical research database of melanoma and cases were carefully documented, (many following strict imaging protocols), we relied on clinical assessments and other test results to ascertain how distant recurrences were first detected. We excluded patients without imaging data or with irregular imaging patterns, which may have introduced bias and which might reduce generalizability. Furthermore, this study was limited to one large, dedicated melanoma center and the survival comparisons presented between each surveillance group may not be generalizable to all settings.

We deliberately excluded patients who developed distant recurrence prior to their first follow-up scan, which may have led to the selection of patients in our cohort with a better prognosis. However, surveillance imaging as part of a follow-up program is unlikely to benefit patients with very early recurrence. We chose to focus on the detection of distant recurrence through surveillance imaging, although many of the patients also developed regional recurrences at some point. Although we recognize there is a possibility that regional melanoma recurrences may be detected through CT or PET/CT imaging, these tumor manifestations are usually able to be detected by physical examination.^23^

Analysis of the impact of surveillance imaging strategies requires consideration of lead-time bias, which occurs when the diagnosis of distant recurrence is made earlier in the more frequent surveillance group, resulting in an apparent increase in survival time from the date of detection of distance recurrence (measured by dMSS). The time from initial melanoma diagnosis to death (measured by MSS and OS) is not subject to lead-time bias given that surveillance imaging commences after the primary diagnosis. Thus, in our analysis of post dMSS, lead-time bias constitutes an artificial addition to the survival time of surveillance-detected distant recurrence. Although correction of lead-time bias will account for the main bias inherent in post dMSS estimation,^24^ randomized trials with survival from the time of original melanoma diagnosis/treatment as an outcome are warranted to establish the existence of this important health benefit. A randomized trial (the TRIM study) has already commenced to determine the impact of imaging on 5-year survival for patients with stage II and III melanoma (https://clinicaltrials.gov/ct2/show/study/NCT03116412).

## Conclusions

More than half of patients with resected stage III melanoma in a follow-up cohort develop distant disease within 5 years, with surveillance imaging detecting a high proportion of these patients. Our study did not demonstrate a survival advantage for patients undergoing more frequent surveillance imaging. Patients undergoing less frequent surveillance had more favorable prognostic characteristics, suggesting that the survival benefits of more frequent imaging, if any, do not override the impact of established prognostic factors and/or better treatments and may be a result of lead-time bias. This study highlights the need for a randomized trial to answer this question. The data presented in this study are fundamental to inform selection of the target population, sample size calculations, choice of the primary outcome measure(s), and feasibility of the study protocol.

## Supplementary Information

Below is the link to the electronic supplementary material.

Supplementary file1 (DOCX 25 KB)
