# Supplementary material for: The Impact of Surveillance Imaging Frequency on the Detection of Distant Disease for Patients with Resected Stage III Melanoma
Source: Ann Surg Oncol. 2022 Feb 10;29(5):2871–81. doi: 10.1245/s10434-021-11231-3 (PMC8990943; doi:10.1245/s10434-021-11231-3)
Supplement: Supplementary file 1 — Supplementary file1 (DOCX 25 KB) [file 10434_2021_11231_MOESM1_ESM.docx]

**Supplementary materials**

**Supplementary figure 1:** Definition of survival outcomes


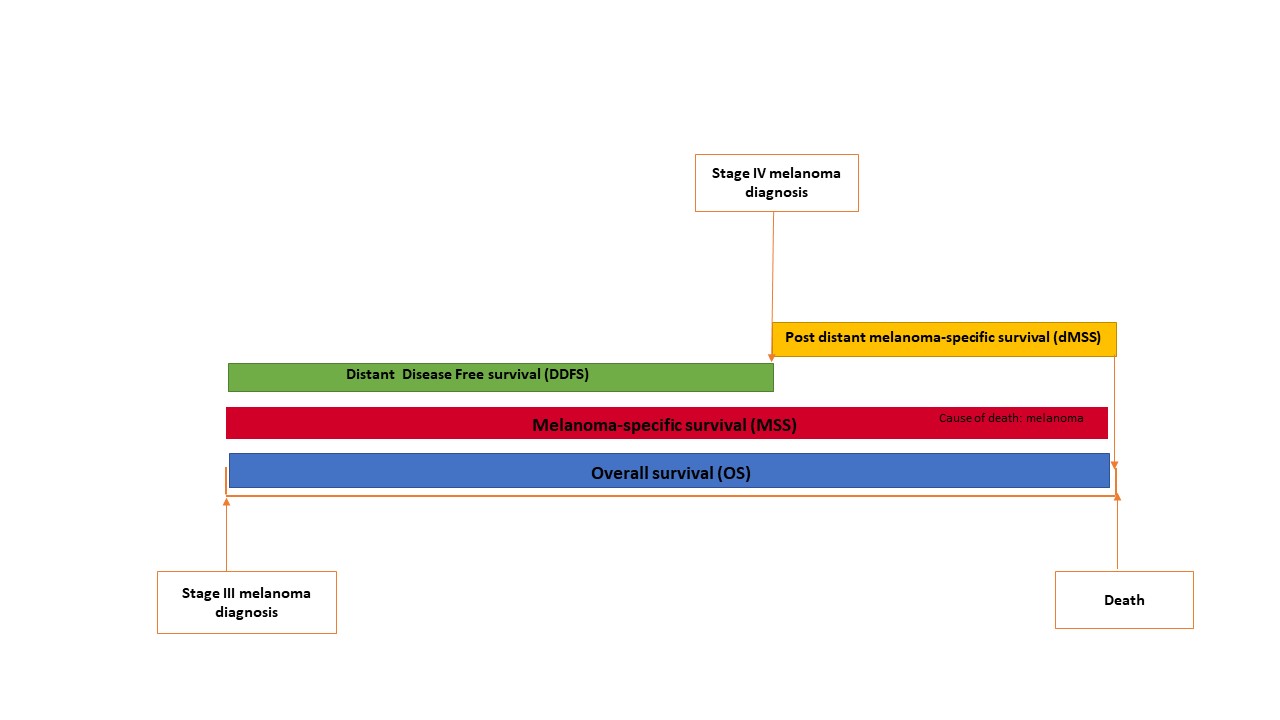


**Supplementary figure 2:** DDFS, MSS and OSS by time (before vs. after 2010)


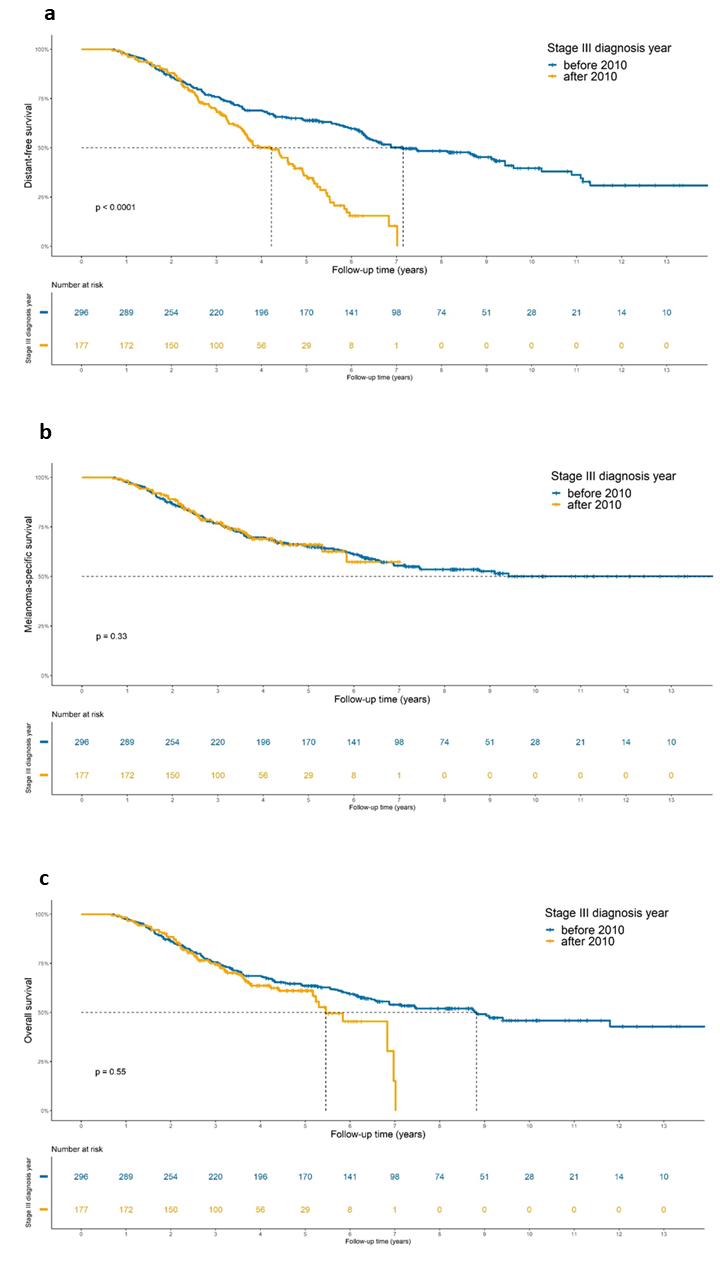


**Supplementary table 1**: Univariate and multivariable Cox regression stratified by Stage (AJCC 8th Ed.) of Distant-disease free survival

|  | | **Univariable** | | **Multivariable** | |
| --- | --- | --- | --- | --- | --- |
| **Variable** |  | **HR** | **P-value** | **HR** | **P-value** |
| **Type folow-up scheduled** | Biannual vs Annual | 1.39 (0.88, 2.19) | 0.154 | 1.69 (1.02, 2.78) | 0.040 |
|  | Intensive vs Annual | 4.72 (3.55, 6.29) | <.001 | 4.57 (3.25, 6.45) | <.001 |
| **Type of investigation** | PET, PET/CT vs | 1.20 (0.83, 1.71) | 0.330 | 1.43 (0.90, 2.27) | 0.130 |
| **Age at stage III diagnosis (every 10-year increase)** |  | 1.12 (1.03, 1.23) | 0.012 | 1.07 (0.97, 1.19) | 0.168 |
| **Sex** | Female vs Male | 0.94 (0.73, 1.22) | 0.667 |  |  |
| **Primary site** | Lower limbs vs | 0.66 (0.44, 0.98) | 0.038 | 0.64 (0.43, 0.97) | 0.035 |
|  | Occult vs Head&Neck | 1.04 (0.68, 1.57) | 0.869 |  |  |
|  | Trunks vs Head&Neck | 0.71 (0.49, 1.02) | 0.067 | 0.67 (0.46, 0.99) | 0.045 |
|  | Upper limbs vs | 0.83 (0.51, 1.34) | 0.440 | 0.68 (0.41, 1.12) | 0.133 |
| **Breslow thickness (mm)** |  | 1.03 (1.00, 1.06) | 0.025 | 1.04 (1.00, 1.07) | 0.047 |
| **Mitotic rate** |  | 1.02 (1.00, 1.04) | 0.051 |  |  |
| **Ulceration** | Yes vs No | 1.11 (0.79, 1.56) | 0.541 |  |  |
| **regression** | Early vs Absent | 0.97 (0.71, 1.32) | 0.833 |  |  |
|  | Intermediate vs Absent | 1.06 (0.56, 2.01) | 0.869 |  |  |
|  | Late vs Absent | 0.76 (0.42, 1.38) | 0.365 |  |  |
|  | Not reported vs | 1.20 (0.64, 2.24) | 0.564 |  |  |
| **SNB performed** | Yes vs No | 0.66 (0.50, 0.86) | 0.002 | 0.86 (0.56, 1.33) | 0.510 |
| **SNB result** | Positive vs Negative | 1.94 (0.85, 4.42) | 0.114 |  |  |
| **LND performed** | Yes vs No | 1.41 (1.07, 1.85) | 0.015 | 1.21 (0.87, 1.68) | 0.251 |
| **LND result** | Positive vs Negative | 1.25 (0.92, 1.69) | 0.159 |  |  |
| **Radiotherapy provided** | Yes vs No | 5.39 (3.94, 7.38) | <.001 |  |  |

**Supplementary table 2**: Univariate and multivariable Cox regression stratified by Stage (AJCC 8th Ed.) of melanoma-specific survival

|  | | **Univariable** | | **Multivariable** | |
| --- | --- | --- | --- | --- | --- |
| **Variable** |  | **HR** | **P-value** | **HR** | **P-value** |
| **Type folow-up scheduled** | Biannual vs Annual | 0.90 (0.48, 1.69) | 0.747 | 1.25 (0.66, 2.40) | 0.495 |
|  | Intensive vs Annual | 5.40 (3.88, 7.52) | <.001 | 5.28 (3.55, 7.87) | <.001 |
| **Type of investigation** | PET, PET/CT vs | 1.16 (0.72, 1.85) | 0.543 |  |  |
| **Age at stage III diagnosis (every 10-year increase)** |  | 1.16 (1.04, 1.30) | 0.007 | 1.05 (0.93, 1.20) | 0.407 |
| **Sex** | Female vs Male | 0.99 (0.73, 1.35) | 0.966 |  |  |
| **Primary site** | Lower limbs vs | 1.16 (0.69, 1.94) | 0.579 | 1.22 (0.71, 2.08) | 0.466 |
|  | Occult vs Head&Neck | 1.65 (0.98, 2.80) | 0.060 |  |  |
|  | Trunks vs Head&Neck | 1.14 (0.70, 1.85) | 0.609 | 1.18 (0.71, 1.96) | 0.517 |
|  | Upper limbs vs | 1.36 (0.75, 2.46) | 0.309 | 1.11 (0.60, 2.04) | 0.733 |
| **Breslow thickness (mm)** |  | 1.04 (1.00, 1.08) | 0.030 | 1.05 (1.00, 1.09) | 0.043 |
| **Mitotic rate** |  | 1.02 (0.99, 1.04) | 0.147 |  |  |
| **Ulceration** | Yes vs No | 1.44 (0.95, 2.20) | 0.087 |  |  |
| **regression** | Early vs Absent | 0.85 (0.59, 1.24) | 0.411 |  |  |
|  | Intermediate vs Absent | 0.66 (0.26, 1.68) | 0.382 |  |  |
|  | Late vs Absent | 0.78 (0.39, 1.54) | 0.472 |  |  |
|  | Not reported vs | 1.11 (0.54, 2.28) | 0.784 |  |  |
| **SNB performed** | Yes vs No | 0.75 (0.54, 1.03) | 0.074 | 0.98 (0.56, 1.71) | 0.952 |
| **SNB result** | Positive vs Negative | 1.41 (0.57, 3.49) | 0.451 |  |  |
| **LND performed** | Yes vs No | 1.21 (0.87, 1.67) | 0.263 | 1.02 (0.69, 1.50) | 0.931 |
| **LND result** | Positive vs Negative | 1.09 (0.75, 1.58) | 0.650 |  |  |
| **Radiotherapy provided** | Yes vs No | 5.86 (3.94, 8.69) | <.001 |  |  |

**Supplementary table 3**: Univariate and multivariable Cox regression stratified by Stage (AJCC 8th Ed.) of Overall-survival

|  | | **Univariable** | | **Multivariable** | |
| --- | --- | --- | --- | --- | --- |
| **Variable** |  | **HR** | **P-value** | **HR** | **P-value** |
| **Type follow-up scheduled** | Biannual vs Annual | 0.89 (0.50, 1.60) | 0.704 | 1.21 (0.65, 2.28) | 0.545 |
|  | Intensive vs Annual | 5.02 (3.66, 6.86) | <.001 | 5.20 (3.53, 7.66) | <.001 |
| **Type of investigation** | PET, PET/CT vs | 1.37 (0.90, 2.09) | 0.140 | 1.19 (0.68, 2.08) | 0.540 |
| **Age at stage III diagnosis (every 10-year increase)** |  | 1.21 (1.09, 1.34) | <.001 | 1.10 (0.98, 1.24) | 0.116 |
| **Sex** | Female vs Male | 0.87 (0.64, 1.17) | 0.351 |  |  |
| **Primary site** | Lower limbs vs | 1.11 (0.68, 1.83) | 0.673 | 1.15 (0.69, 1.93) | 0.591 |
|  | Occult vs Head&Neck | 1.64 (0.99, 2.71) | 0.054 |  |  |
|  | Trunks vs Head&Neck | 1.21 (0.76, 1.93) | 0.415 | 1.26 (0.78, 2.04) | 0.344 |
|  | Upper limbs vs | 1.29 (0.73, 2.29) | 0.384 | 1.04 (0.57, 1.87) | 0.908 |
| **Breslow thickness (mm)** |  | 1.04 (1.01, 1.08) | 0.017 | 1.05 (1.01, 1.09) | 0.027 |
| **Mitotic rate** |  | 1.02 (1.00, 1.04) | 0.070 |  |  |
| **Ulceration** | Yes vs No | 1.42 (0.96, 2.12) | 0.081 |  |  |
| **regression** | Early vs Absent | 0.92 (0.64, 1.31) | 0.641 |  |  |
|  | Intermediate vs Absent | 0.77 (0.34, 1.74) | 0.535 |  |  |
|  | Late vs Absent | 0.80 (0.42, 1.55) | 0.510 |  |  |
|  | Not reported vs | 1.13 (0.57, 2.26) | 0.720 |  |  |
| **SNB performed** | Yes vs No | 0.76 (0.56, 1.03) | 0.075 | 0.96 (0.57, 1.64) | 0.894 |
| **SNB result** | Positive vs Negative | 1.19 (0.55, 2.59) | 0.656 |  |  |
| **LND performed** | Yes vs No | 1.22 (0.90, 1.67) | 0.202 | 1.03 (0.71, 1.49) | 0.889 |
| **LND result** | Positive vs Negative | 1.25 (0.88, 1.77) | 0.220 |  |  |
| **Radiotherapy provided** | Yes vs No | 4.97 (3.45, 7.15) | <.001 |  |  |

**Supplementary table 4:** Baseline Characteristics stratified by year of stage III diagnosis (2010 vs after 2010)

| **Characteristics** | **Before 2010 (N = 296)** | **After 2010 (N = 177)** | **P-value** |
| --- | --- | --- | --- |
| **Type of FU schedule** |  |  |  |
| Annual | 196 (66.2%) | 89 (50.3%) | 0.0028 |
| Biannual | 25 (8.4%) | 22 (12.4%) |  |
| Intensive | 75 (25.3%) | 66 (37.3%) |  |
| **Type of investigation** |  |  |  |
| CT | 281 (94.9%) | 120 (67.8%) | <.0001 |
| PET, PET/CT | 15 (5.1%) | 57 (32.2%) |  |
| **AJCC 8th Ed. Stage** |  |  |  |
| IIIa | 61 (20.6%) | 28 (15.8%) | 0.3673 |
| IIIb | 93 (31.4%) | 53 (29.9%) |  |
| IIIc | 139 (47.0%) | 92 (52.0%) |  |
| IIId | 3 (1.0%) | 4 (2.3%) |  |
| **Age at stage III diagnosis (years)** |  |  |  |
| Mean (sd) | 52.4 (14.6) | 57.9 (14.6) | <.0001 |
| Median (range) | 52.0 (19.0, 84.0) | 59.0 (21.0, 89.0) |  |
| **Sex** |  |  |  |
| Male | 190 (64.2%) | 113 (63.8%) | 0.9393 |
| Female | 106 (35.8%) | 64 (36.2%) |  |
| **Primary site** |  |  |  |
| Head&Neck | 46 (15.5%) | 28 (15.8%) | 0.0421 |
| Trunks | 80 (27.0%) | 70 (39.5%) |  |
| Upper limbs | 32 (10.8%) | 18 (10.2%) |  |
| Lower limbs | 76 (25.7%) | 37 (20.9%) |  |
| Occult | 62 (20.9%) | 24 (13.6%) |  |
| **Breslow thickness (mm)** |  |  |  |
| Mean (sd) | 3.5 (3.0) | 4.4 (5.2) | 0.0303 |
| Median (range) | 2.6 (0.5, 28.0) | 2.8 (0.0, 40.0) |  |
| **Mitotic rate** |  |  |  |
| Mean (sd) | 6.3 (5.9) | 8.8 (8.9) | 0.0013 |
| Median (range) | 5.0 (0.0, 45.0) | 5.0 (0.0, 57.0) |  |
| **Ulceration** |  |  |  |
| No | 137 (60.1%) | 84 (56.8%) | 0.5215 |
| Yes | 91 (39.9%) | 64 (43.2%) |  |
| **Regression** |  |  |  |
| Absent | 81 (34.6%) | 53 (34.6%) | 0.9817 |
| Early | 105 (44.9%) | 72 (47.1%) |  |
| Intermediate | 13 (5.6%) | 7 (4.6%) |  |
| Late | 21 (9.0%) | 12 (7.8%) |  |
| Not reported | 14 (6.0%) | 9 (5.9%) |  |
| **SNB performed** |  |  |  |
| No | 85 (28.7%) | 58 (32.8%) | 0.3531 |
| Yes | 211 (71.3%) | 119 (67.2%) |  |
| **SNB result** |  |  |  |
| Negative | 13 (6.2%) | 4 (3.4%) | 0.2692 |
| Positive | 198 (93.8%) | 115 (96.6%) |  |
| **LND performed** |  |  |  |
| No | 104 (35.1%) | 51 (28.8%) | 0.1564 |
| Yes | 192 (64.9%) | 126 (71.2%) |  |
| **LND result** |  |  |  |
| Negative | 102 (53.1%) | 63 (50.0%) | 0.5854 |
| Positive | 90 (46.9%) | 63 (50.0%) |  |
| **Total number of positive nodes** |  |  |  |
| Mean (sd) | 2.1 (3.7) | 2.2 (3.0) | 0.6855 |
| Median (range) | 1.0 (0.0 43.0) | 1.0 (0.0 33.0) |  |
| **Method of diagnosis of recurrence** |  |  |  |
| CT | 35 (20.3%) | 25 (22.5%) | 0.1182 |
| PET | 12 (7.0%) | 18 (16.2%) |  |
| PET/CT | 3 (1.7%) | 4 (3.6%) |  |
| Clinical | 0 (0.0%) | 1 (0.9%) |  |
| FNAB (cytology) | 1 (0.6%) | 1 (0.9%) |  |
| Hispathology | 60 (34.9%) | 29 (26.1%) |  |
| Cytology/Haematology | 27 (15.7%) | 11 (9.9%) |  |
| Other imaging (MRI, Xray, Ultrasound) | 34 (19.8%) | 22 (19.8%) |  |
| **Radiotherapy provided** | | |  |
| No | 164 (64.6%) | 88 (66.7%) | 0.6810 |
| Yes | 90 (35.4%) | 44 (33.3%) |  |
